# Whole exome sequencing in 342 congenital cardiac left sided lesion cases reveals extensive genetic heterogeneity and complex inheritance patterns

**DOI:** 10.1186/s13073-017-0482-5

**Published:** 2017-10-31

**Authors:** Alexander H. Li, Neil A. Hanchard, Dieter Furthner, Susan Fernbach, Mahshid Azamian, Annarita Nicosia, Jill Rosenfeld, Donna Muzny, Lisa C. A. D’Alessandro, Shaine Morris, Shalini Jhangiani, Dhaval R. Parekh, Wayne J. Franklin, Mark Lewin, Jeffrey A. Towbin, Daniel J. Penny, Charles D. Fraser, James F. Martin, Christine Eng, James R. Lupski, Richard A. Gibbs, Eric Boerwinkle, John W. Belmont

**Affiliations:** 1 Human Genetics Center, University of Texas Health Science Center, Houston, TX USA; 2 Department of Molecular and Human Genetics, Baylor College of Medicine, Houston, TX USA; 3 Department of Paediatrics, Children’s Hospital, Krankenhausstr. 26-30, 4020 Linz, Austria; 4 Human Genome Sequencing Center, Baylor College of Medicine, Houston, TX USA; 5 Department of Pediatrics, Baylor College of Medicine, Houston, TX USA; 6 Division of Cardiology, Seattle Children’s Hospital, Seattle, WA USA; 7 Pediatric Cardiology, University of Tennessee Health Science Center, Memphis, TN USA; 8 Department of Surgery, Baylor College of Medicine, Houston, TX USA; 9 Department of Molecular Physiology and Biophysics, Baylor College of Medicine, and the Texas Heart Institute, Houston, TX USA; 10 Texas Children’s Hospital, Houston, TX USA; 11 5200 Illumina Way, San Diego, CA USA

**Keywords:** Congenital heart disease, Cardiac malformation, Developmental disorder, Rare disease, Whole exome sequence

## Abstract

**Background:**

Left-sided lesions (LSLs) account for an important fraction of severe congenital cardiovascular malformations (CVMs). The genetic contributions to LSLs are complex, and the mutations that cause these malformations span several diverse biological signaling pathways: TGFB, NOTCH, SHH, and more. Here, we use whole exome sequence data generated in 342 LSL cases to identify likely damaging variants in putative candidate CVM genes.

**Methods:**

Using a series of bioinformatics filters, we focused on genes harboring population-rare, putative loss-of-function (LOF), and predicted damaging variants in 1760 CVM candidate genes constructed a priori from the literature and model organism databases. Gene variants that were not observed in a comparably sequenced control dataset of 5492 samples without severe CVM were then subjected to targeted validation in cases and parents. Whole exome sequencing data from 4593 individuals referred for clinical sequencing were used to bolster evidence for the role of candidate genes in CVMs and LSLs.

**Results:**

Our analyses revealed 28 candidate variants in 27 genes, including 17 genes not previously associated with a human CVM disorder, and revealed diverse patterns of inheritance among LOF carriers, including 9 confirmed de novo variants in both novel and newly described human CVM candidate genes (*ACVR1*, *JARID2, NR2F2*, *PLRG1, SMURF1)* as well as established syndromic CVM genes (*KMT2D*, *NF1*, *TBX20*, *ZEB2*). We also identified two genes (*DNAH5*, *OFD1*) with evidence of recessive and hemizygous inheritance patterns, respectively. Within our clinical cohort, we also observed heterozygous LOF variants in *JARID2* and *SMAD1* in individuals with cardiac phenotypes, and collectively, carriers of LOF variants in our candidate genes had a four times higher odds of having CVM (odds ratio = 4.0, 95% confidence interval 2.5–6.5).

**Conclusions:**

Our analytical strategy highlights the utility of bioinformatic resources, including human disease records and model organism phenotyping, in novel gene discovery for rare human disease. The results underscore the extensive genetic heterogeneity underlying non-syndromic LSLs, and posit potential novel candidate genes and complex modes of inheritance in this important group of birth defects.

**Electronic supplementary material:**

The online version of this article (doi:10.1186/s13073-017-0482-5) contains supplementary material, which is available to authorized users.

## Background

Congenital cardiovascular malformations (CVMs) occur in 5 to 8 of 1000 live births and have a high mortality rate compared to other birth defects [1, 2]. Left-sided lesion (LSL) disorders comprise 15–20% of severe CVMs [3, 4] and include hypoplastic left heart syndrome (HLHS), aortic valve stenosis (AS), coarctation of the aorta (CoA), interrupted aortic arch type A (IAAA), mitral valve atresia and stenosis (MA, MS), and Shone’s complex (SC). Despite the diversity of the cardiac malformations encompassed by LSLs, this epidemiologically grouped family is also thought to be developmentally unified by altered or obstructed flow through the left side of the heart during embryonic development [5]. Most importantly, LSLs, and HLHS in particular, contribute disproportionately high costs to long-term disability and mortality from CVM, making a better understanding of their underlying etiology an important area of study.

Although genetic factors are known to contribute significantly to the development of LSLs, the spectrum and nature of these genetic contributions are complex and heterogeneous, spanning single nucleotide substitutions, chromosome abnormalities including aneuploidies [6], structural variants including copy number variants (CNVs) causing genomic disorders [7], and oligogenic inheritance [8]. More than 30 genes have been implicated in human syndromes that include left-sided heart malformations. These loci include those implicated in HLHS (*ZIC3*, *TBX5*, *CREBBP*, *ACVR2B*, *LEFTY2*, *DTNA*, *DHCR7*, *EVC1-2*, *FOXF1*-*FOXC2*-*FOXL1*, and *PEX* genes), AS (*NOTCH1, FOXC1*, *FGD1*), and CoA (*JAG1*, *NOTCH2*, *NF1*, *PTPN11*, *KRAS*, *SOS1*, *RAF1*, *NRAS*, *BRAF*, *SHOC2*, *CBL*, *ZIC3*, *CREBBP*, *MLL2*, *FGD1*, *DHCR7*, *NSDHL*, *KCNJ2*, *MKS1*) (Additional file 1: Table S1). Many of the associated syndromes have characteristic extra-cardiac features indicating early pleiotropic roles for the underlying molecular pathways in normal organ development. LSLs without overt extra-cardiac abnormalities (apparently isolated or non-syndromic LSL) appear to have a more complex origin. Familial clustering of cases [9] and an increased risk of LSL in first-degree relatives [10] are consistent with single gene and/or oligogenic inheritance; however, the consistent observation of sporadic cases, particularly in the context of more severe LSLs (e.g., HLHS) that may negatively impact reproductive fitness, suggests a potential role for de novo mutations and a potential role for multi-locus variation [11]. De novo mutations have been reported within the broader context of congenital heart defects (CHDs) [12, 13], but their specific role in LSLs remains unknown.

**Fig. 1:**
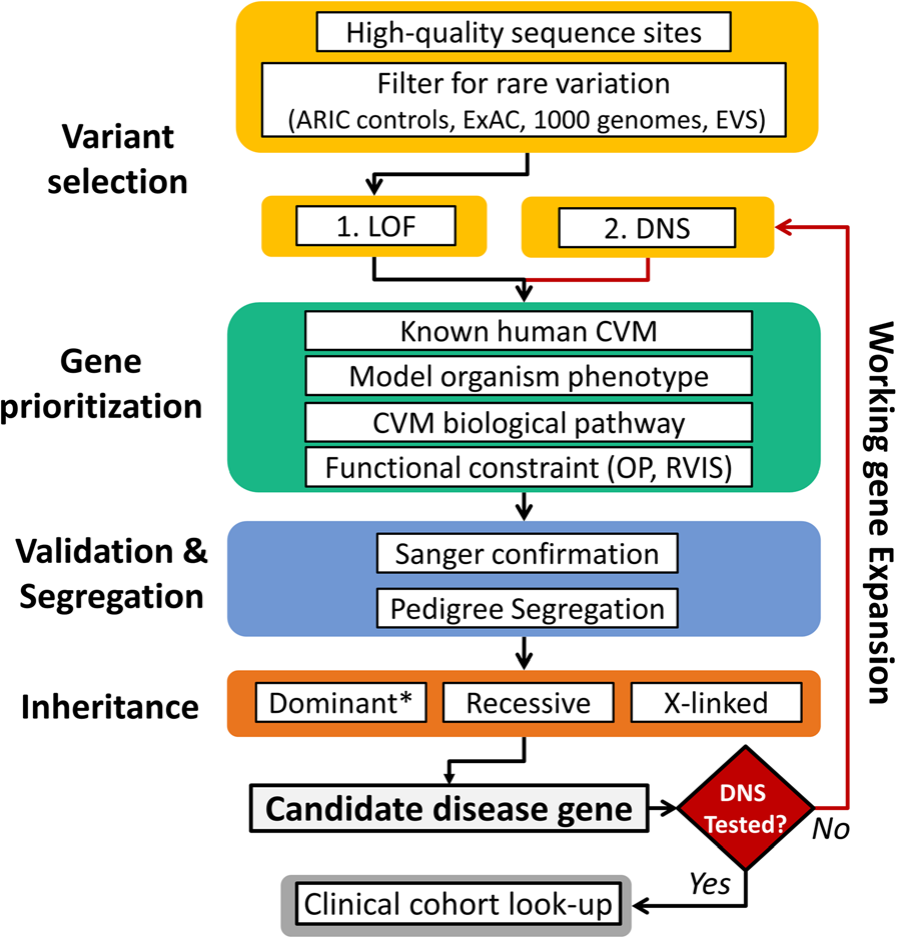
Discovery strategy for LSL cohort. Imposing a candidate list, constructed independently, of a priori disease gene candidates on rare-variant exome-wide analyses, with integration of pedigree information, facilitates genes discovery. *LOF* putative loss-of-function variants, *DNS* damaging non-synonymous variation predicted by > 3 of 6 predictive algorithms, *ARIC* Atherosclerosis Risk in Communities, ExAC Exome Aggregation Consortium, *EVS* Exome Variant Server, *OP* ratio of observed to potential LOF alleles, *RVIS* residual variation intolerance score; *includes de novo and inherited dominant alleles

This confluence of multiple disease models, potential modes of inheritance, and disparate candidate genes presents a challenge for identifying likely disease-causing alleles in LSLs. The availability of large-scale, public databases that aid in annotating and curating extensive genomic data offers a lens through which to focus on the most robust CVM candidate genes. In order to gain a deeper understanding of the spectrum of genetic variation associated with LSLs, we performed whole exome sequencing (WES) of 342 unrelated LSL cases without known extra-cardiac features, i.e., apparent non-syndromic LSLs. We then focused on putative loss-of-function (LOF) or predicted damaging variants rarely observed in public databases and not observed in a large cohort not enriched for LSLs that was sequenced on a similar platform. We then applied a series of bioinformatic filters to a standing list of potential CHD candidate genes derived from the literature and publicly available databases in order to focus the set of WES-derived genomic candidates. The intersection of case-exclusive, rare, putatively damaging, variation with this a priori LSL candidate gene list formed the foundation of our discovery strategy (Fig. 1). Candidate variants identified were then validated and genotyped in available parents to determine patterns of inheritance. Genes with verified de novo variants were then queried for variants with LOF consequences and algorithmically damaging variants in a large clinical database consisting of individuals with developmental concerns referred for clinical sequencing.

## Methods

### Subject selection

The discovery sample included 342 unrelated LSL cases without clinically evident extra-cardiac malformations or unexplained developmental concerns at the time of recruitment, which in most cases was during infancy or early childhood. Cases were recruited through the Texas Children’s Hospital (TCH) in Houston, TX and included 42 cases with HLHS originally recruited at Children’s Hospital in Linz, Austria. Parents and affected family members of LSL cases (where available) were also recruited. TCH participants were recruited as an extension of a previously published [9] cohort of LSL cases, and all participants provided informed consent prior to their inclusion. In brief, patients were eligible if they had a characteristic LSL CVM, — AS, CoA, HLHS, MA or MS — or a combination of aortic valve atresia or stenosis with hypoplasia of the left ventricle and aortic arch known as Shone’s complex (SC). We also included individuals if they had bicuspid aortic valve (BAV), ventricular septal defect (VSD), or other associated cardiac defects when they were present in combination with typical LSL malformations (Table 1). Diagnoses were confirmed by echocardiography, cardiac catheterization, or open cardiac surgery. Cases with clear evidence of extra-cardiac involvement at the time of evaluation by the referring physician (including dysmorphic or syndromic cases or those with other birth defects or congenital anomalies) were excluded. In some cases, particularly among medically unstable neonates, a comprehensive physical examination was not possible, and cases were included as long as there was not a strong clinical suspicion of a syndromic diagnosis. All cases were genotyped by chromosome microarray as part of a genome-wide association study of the LSL phenotype [14]. Cases found to have large (> 1 megabase) genomic defects were excluded from further analysis [15]. The same single nucleotide polymorphism (SNP) genotype data were also used to infer ancestry from the first two principal components on multi-dimensional scaling (MDS).

**Table 1:** Overview of LSL cases

Category	Description	Cases (*n*)
Ancestry	African American	1
	Caucasian	246
	Hispanic	95
Sex	Male	233
	Female	108
CVM	Aortic valve stenosis (AS)	63
	Coarctation of the aorta (CoA)	130
	Hypoplastic left heart syndrome (HLHS)	136
	Mitral valve stenosis (MS)	2
	Shone’s complex (SC)	6
	Other	4

This table summarizes demographic and clinical information of 342 LSL probands

*CVM* cardiovascular malformation

### Secondary cohorts

Our analysis utilized two other cohorts as part of our approach. First, we used WES data from 5492 European American (EA) individuals from the population-based Atherosclerosis Risk in Communities (ARIC) study [16] as a comparison group without known severe LSLs or significant congenital CVMs. ARIC WES data [17] were generated on the same sequencing platform using a similar capture reagent and were annotated in the same way as the LSL cohort (see below). The ARIC data were primarily used as a sequencing control dataset to reduce the likelihood of case-only variants arising because of differences in sequencing platforms in public databases. ARIC samples with any of the following criteria were excluded from these analyses: heart failure, major Q-wave, or left ventricular hypertrophy (LVH) by the Cornell definition. In the second stage, genes with de novo LOF variation ascertained in the discovery stage were interrogated for similar damaging/LOF variants in an independent clinical sample of 4750 clinical exomes from the Baylor Genetics Laboratory (http://www.bmgl.com). These individuals had samples submitted to the laboratory for clinical WES for a diverse set of clinical indications, including children with neurodevelopmental concerns or congenital birth defects in keeping with previous reports from this cohort [18, 19]. Only the primary indication and reported clinical data were available for interrogation; cardiovascular lesions, where indicated, were noted, but were not systematically assessed.

### Whole exome sequencing

WES was performed on cases and comparison samples with the Illumina HiSeq platform using the Mercury pipeline [20]. ARIC samples were captured using VCRome 2.1 (42 Mb) reagents with an average coverage of 88×, LSL cases were captured using the Human Genome Sequencing Center (HGSC) core (52 Mb), and all analyses were restricted to exonic regions shared between these two reagents. Overall exon coverage and read depth in the two cohorts were highly comparable (> 90% of samples with ≥ 20× coverage at shared sites). In both cohorts, read mapping to Genome Reference Consortium Human Build 37 (GRCh37) was performed with Burrows-Wheeler alignment [21], and allele calling was performed with the Atlas2 suite (Atlas-SNP, Atlas-Indel) [22]. The Variant Call Format (VCF) file contained flagged low-quality variants including SNPs with posterior probability lower than 0.95, total depth of coverage less than 10×, fewer than three variant reads, allelic fraction less than 10%, 99% reads in a single direction, and homozygous reference alleles with < 6× coverage. We increased stringency to remove low-quality indels with a total depth less than 30× and allelic fraction below 30%.

WES of LSL cases initially revealed 243,609 variants within the capture regions (239,726 single nucleotide substitutions and 3883 small indels), with indel length ranging from –51 bp (deletion) to +26 bp (insertion). On average, each case presented a total of 14,669 heterozygous and 8321 homozygous non-reference genotypes (Additional file 2: Table S2). Population frequency of variants was determined by comparison to the 1000 Genomes Project [23], Exome Sequencing Project, Exome Aggregation Consortium (ExAC) v0.3 [24], and participants from the ARIC study [16]. Only novel and rare sites were included in these analyses, defining rare as minor allele frequency (MAF) < 0.5% for LOF dominant, recessive, and X-linked segregation analyses.

### Functional variant prediction

Variants were annotated to RefSeq gene definitions using ANNOVAR [25]. Conservative LOF annotation was performed by selecting only included premature stopgains in the non-terminal exon, variants disrupting essential splice sites used by all gene isoforms, and frameshift indels similarly mapping to all isoforms. Damaging non-synonymous (DNS) variation was defined as protein-altering substitutions predicted to be damaging by a consensus of at least three out of six prediction scores downloaded via dbNSFP [26] (SIFT, Polyphen2 HDIV, LRT, MutationTaster, MutationAssessor, FATHMM). A Phred-like scaled C-score (Combined Annotation Dependent Depletion (CADD) [27]) was also used to assess pathogenicity of variants (LOF and DNS) but was not used to exclude candidate sites. Residual variation intolerance scores (RVISs) [28] for genes were also interrogated as a means of further prioritizing likely candidate genes.

### A priori gene prioritization

In order to facilitate novel gene and variant discovery, we assembled a priori evidence from public resources to identify potential novel LSL genes. We compiled a list of 1712 human genes with a putative role in the development of CVM from a variety of public resources (Additional file 3: Table S3). Genes related to overlapping human disorders including CVM were ascertained from the National Center for Biotechnology Information (NCBI), Online Mendelian Inheritance in Man (OMIM), and literature searches. Relevance to biological pathways and interactions (*SHH*, *NOTCH*, *TGFB*, *PITX*) was determined using the Kyoto Encyclopedia of Genes and Genomes (KEGG) database [29]. Two model organism databases, the Zebrafish Information Network (ZFIN [30]) and Mouse Genome Informatics (MGI [31]), were also used to ascertain genes related to cardiac malformation in model organisms. ZFIN was queried for genes expressed in the zebrafish heart, and MGI was queried for genes causing abnormal cardiac morphology in mouse models (MP:0000266).

### Web resources

Web resources utilized in the study are provided below:

1000 Genomes, http://grch37.ensembl.org/index.html

ExAC Browser, http://exac.broadinstitute.org/

dbNSFP, http://varianttools.sourceforge.net/Annotation/DbNSFP

OMIM, http://www.omim.org/

University of California Santa Cruz (UCSC) Genome Browser, http://genome.ucsc.edu

HMZDelfinder, https://github.com/BCM-Lupskilab/HMZDelFinder

ClinVar, https://www.ncbi.nlm.nih.gov/clinvar/

Human Gene Mutation Database (HGMD), http://www.hgmd.cf.ac.uk/ac/index.php

### Analytical approach

Candidate mutations in the LSL cohort were prioritized using two main criteria: (1) extremely low allele frequency compared to that observed in the ARIC database (minor allele < 0.05%) and public databases (1000 Genomes Project, ESP, and ExAC; minor allele < 0.5%); (2) prediction of a deleterious functional effect including LOF and DNS variation. Initially, we focused on the most damaging class of variation — extremely rare LOF — and conservatively omitted recurrent LOF sites (seen more than two times) due to concerns about potential ethnic stratification. We further enriched for genes likely to contribute to LSLs by quantifying the observed LOF constraint of genes in ARIC. First, for each gene, we counted the number of LOF alleles in ARIC (gene-wise observed LOF). Next, we simulated all potential nucleotide substitutions in exonic regions to determine the number of total potential LOF sites for each gene, and calculated the ratio of observed to potential LOF alleles (OP ratio [32]). Genes with a very low OP ratio (zero, or in the lowest 30^th^ percentile) were considered stronger candidates for disease. In general, OP ratios correlated well with the functional gene constraint (pLI) metric in the Exome Aggregation Consortium Database (Table 1). Finally, we filtered for variants occurring in our set of a priori compiled cardiac genes (Additional file 3: Table S3) in order to identify those with supporting evidence for a role in LSLs or CHDs. Once we had ascertained a set of candidate genes with LOF variation, we next performed a “working gene” expansion to include DNS sites in the same genes. All prioritized variants were validated using an orthogonal platform (dideoxy-Sanger sequencing), and where parental samples for surveyed probands were available, these samples were used to assess Mendelian segregation of the variant: de novo vs inherited dominant (heterozygous variants), recessive (homozygous or compound-heterozygous variants in *trans* in a given gene), or X-linked (maternally inherited hemizygous variants on the X chromosome). Finally, having defined a set of candidate genes, we interrogated our clinical database for similarly damaging variants in the same genes and compiled a similar list from publicly available clinical sequencing repositories. The analytical strategy is summarized in Fig. 1.

## Results

### Variant prioritization

WES of LSL cases identified 132,182 (129,329 single nucleotide variants (SNVs), 2853 indels) novel or extremely rare (MAF < 0.5%) variants within the LSL cases. In silico ‘functional annotation’ determined that 4161 of these rare/novel sites were predicted LOF variants (1469 premature stop, 602 splice, and 2090 frameshift, MAF < 0.5%) representing 1660 genes, and 34,100 predicted DNS variants from 11,822 genes. Novel candidate variants for LSLs were subject to stricter, computationally derived, functional criteria and lower frequency thresholds in population comparison groups (Methods). The mean number of rare variants per LSL case after filtering was 54.4 LOF and 118.8 DNS (range LOF = 35–74; range DNS = 88–158, Additional file 4: Figure S1). The intersection of our a priori candidate gene list with validated WES variants from these LSL cases revealed 27 genes harboring rare or case-exclusive LOF alleles, which we prioritized for further study. These alleles were observed in 26 cases (one case presented with two distinct variants, Table 2), or 7.6% (26/342) of our starting cohort, which we next assessed for mode of inheritance (Fig. 1).

**Table 2:** Discovery genes ascertained via case-exclusive LOF sites with evidence for a role in LSLs

Gene	Chr.	LSL		Clinical cohort		Gene support	LOF OP (%ile)	pLI
		LSL ID	Mode	CVM	Non-CVM			
*ACVR1*	2	LO1462	De novo	0	0	MGI, HE, TGFB	0.201	0.96
*JARID2*	6	LO0189	De novo	1	0	MGI, PITX2	0	0.99
*KMT2D*	12	LO0785	De novo	7	13	CVM	0	1
*NF1*	17	LO2000	De novo	1	4	CVM, MGI, HE	0.166	1
*NR2F2*	15	LO0260	De novo	0	0	CVM, MGI	0	0.91
*PLRG1*	4	LO0943	De novo	0	0	MGI	0	0.99
*SMURF1*	7	LO1765	De novo	1	0	TGFB	0	0.99
*TBX20*	7	LO0746	De novo	0	0	CVM, MGI, HE	0	0.34
*ZEB2*	2	LO0747	De novo	6	4	CVM, ZFIN	0	0.99
*ARHGEF11*	1	LO2218	Inherited	2	0	ZFIN	0.156	0.99
*CCDC91*	12	LO0222	Inherited	1	3	PITX2	0	3.2E-05
*CDH2*	18	LO1263	Inherited	2	2	MGI, HE, PITX2	0.077	1
*E2F6*	2	LO0970	Inherited	0	0	PITX2	0	0.55
*FGF19*	11	LO1957	Inherited	0	0	MGI	0	0.29
*GJC1*	17	LO0192	Inherited	0	0	MGI, ZFIN	0	0.94
*GLRX3*	10	LO0350	Inherited	0	0	MGI, HE	0	0.63
*LATS2*	13	LO0238	Inherited	2	4	MGI	0.119	0.87
*LTBP1*	2	LO2218	Inherited	1	2	MGI, TGFB	0	0.53
*MNDA*	1	LO0369	Inherited	0	0	CHD candidate	0.265	7.2E-11
*PCDHGA2*	5	LO0605	Inherited	0	4	HE	0	8.2E-07
*PCSK6*	15	LO1210	Inherited	2	3	MGI	0	0.01
*RAC1*	7	LO0938	Inherited	0	0	MGI, HE	0	0.57
*DNAH5*	5	LO1298	Recessive	6	30	CVM, MGI, HE, PCD	0.299	5.8E-37
*OFD1*	X	LO1488	X-linked	1	3	CVM, MGI, ZFIN	0	0.98
*BMP1*	8	LO0605	Unknown	0	1	MGI	0	0.87
*JMJD6*	17	LO0295	Unknown	0	1	MGI	0	0.82
*ROCK1*	18	LO0453	Unknown	1	5	MGI, TGFB, PITX2	0	1

Inheritance was determined by Sanger sequencing in cases and parents. The number of samples in the clinical laboratory cohort with any LOF alleles in these genes is also provided along with current CVM status. Unknown inheritance indicates only one parent was available for validation and did not carry the mutation

Gene support symbols are defined as follows: *CVM* known role in human cardiovascular malformation, *MGI* overlapping phenotype in mouse, *HE* human heart expressed, *PITX2* related to PITX2 transcription, *ZFIN* overlapping phenotype in zebrafish, TGFB transforming growth factor beta pathway

LOF OP ratio percentile calculated from ARIC EA participants. Functional gene constraint values (pLI) from the Exome Aggregation Consortium (ExAC) Browser (cleaned_exac_r03_march16_z_pli_rec_null_data) are provided for reference

### Discovery genes

Sanger sequencing of these 27 alleles in parents revealed nine to have arisen de novo, all in different genes (*ACVR1*, *JARID2*, *KMT2D*, *NF1*, *NR2F2*, *PLRG1, SMURF1, TBX20*, and *ZEB2*) (Table 2). Mutations in *KMT2D* (MIM 147920), *NF1* (MIM 162200), and *ZEB2* (MIM 235730) are known to cause human monogenic Mendelian syndromes (Kabuki syndrome, neurofibromatosis type 1, and Mowat-Wilson syndrome, respectively), and cardiac malformations in these syndromes occur in 3–50% of patients. Mutations in *NR2F2* (MIM 615779) and *TBX20* (MIM 611363) have previously been associated with non-syndromic congenital heart defects [33, 34]. The remaining de novo alleles were found in genes that are not yet well established as human CVM genes, but are known to play a role in critical cardiac development pathways (e.g., TGFbeta signaling: *SMURF1*, *ACVR1*; *PITX2* transcription factor target: *JARID2* [35]). Model organism mutants recapitulate cardiac developmental phenotypes in seven of these de novo genes (see MGI in Table 2); including *PLRG1*, for which mutant alleles cause malformation of the left ventricle in mouse models [36]. When we expanded our evaluation of these genes to include rare/novel DNS variation (Fig. 1), we identified three additional de novo DNS variants in three genes (*KMT2D, TBX20, ZEB2*), providing further confirmation of their role in LSLs (Additional file 5: Table S4). Outside of de novo variants, the majority of LOF variants observed in our isolated LSL cohort were found in the heterozygous state and were transmitted from an apparently unaffected parent (Table 2, Additional file 5: Table S4); however, we did not perform echocardiograms in parents, leaving open the possibility of incomplete penetrance or variable expressivity of the phenotype, such as BAV and other asymptomatic cardiac anomalies. In addition, we did not systematically investigate for potential mosaicism in the unaffected parent.

We also found evidence for recessive trait inheritance (i.e., homozygous or compound heterozygous alleles) and X-linked inheritance of LOF variants in *DNAH5* and *OFD1,* respectively*.* A homozygous LOF variant was observed in *DNAH5*, which is a reported cause of primary ciliary dyskinesia (MIM 608644), an autosomal recessive condition that may involve complex cardiac malformations in a fraction of patients [37]. We also observed an LOF variant in *OFD1* (X-linked) that was maternally transmitted to an affected male case. Pathologic variation in *OFD1* is the cause of orofaciodigital syndrome (MIM 311200), which can include congenital heart malformations within its phenotypic spectrum [38]. Upon re-evaluation, the individual with this variant did not manifest other features of this syndrome. These observations support recessive and sex-linked forms of LSL and highlight the complex genetic mechanisms underlying these malformations.

**Fig. 2:**
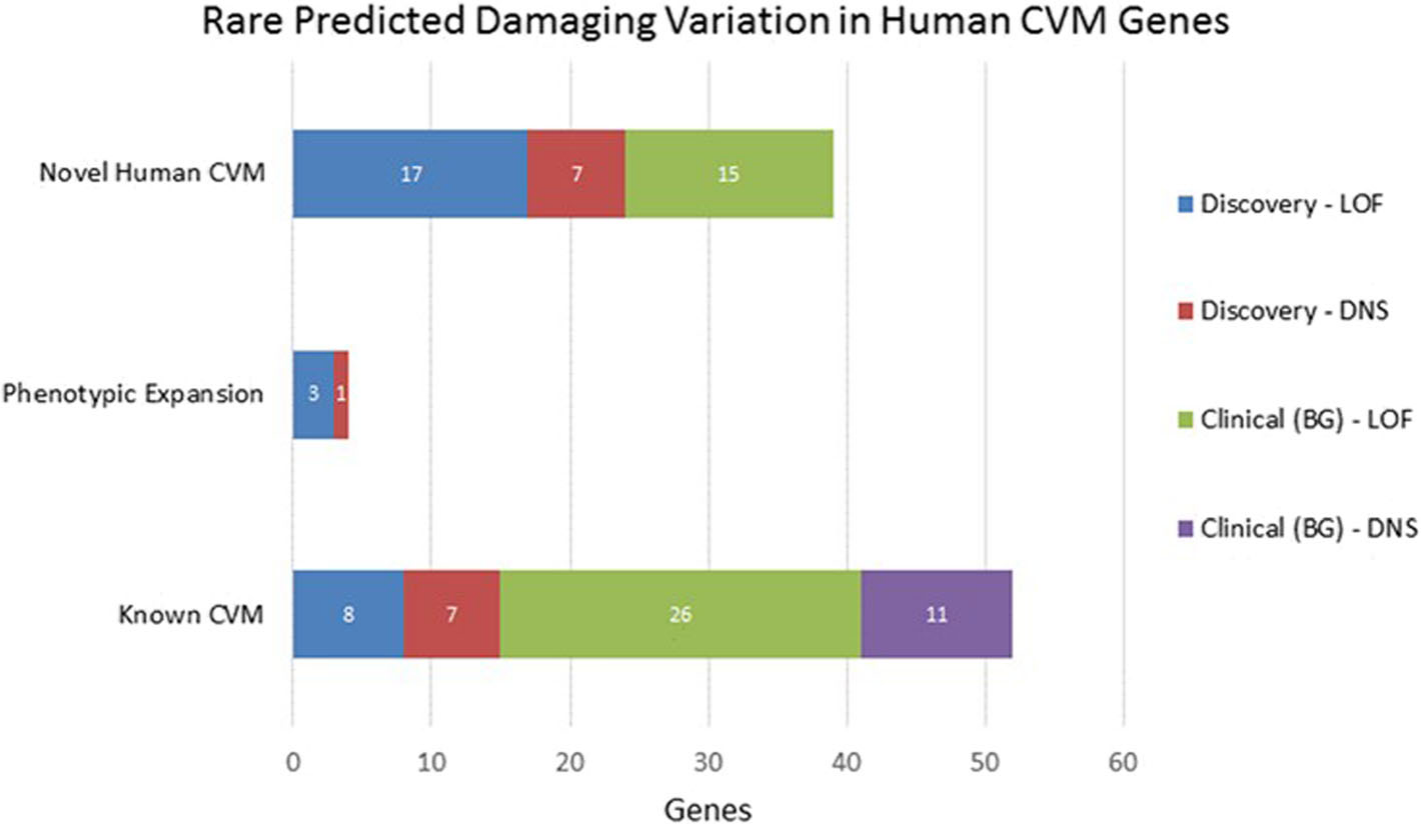
Rare predicted damaging variation in known and novel human cardiovascular malformation (*CVM*) genes. The *x*-axis describes counts of CVM cases carrying predicted loss-of-function (*LOF*) and damaging non-synonymous (*DNS*) variation with observed population frequency < 0.0005. CVM cases include LSL discovery (*n* = 342) and clinical cases referred to Baylor Genetics Lab (*BG*) presenting with cardiac malformations (Additional file 6: Table S5). *Known CVM* indicates counts of cases with variants in genes previously implicated with human CVM in OMIM; *Phenotypic Expansion* indicates genes associated with a human disorder not previously associated with CVM; *Novel Human CVM* genes have not previously been associated with human CVM but were ascertained by our candidate gene strategy (Additional file 3: Table S3)

Having ascertained 27 LSL candidate genes from our a priori candidate list, we then sought to utilize our clinical diagnostic exome sequencing database (Baylor Genetics; see Methods) to provide additional evidence for likely pathogenicity among these genes. In the first instance, we aimed to characterize the relationship between LOF variation in our identified LSL candidate genes and the presence of CVM among clinical cases. This analysis revealed 79 individuals with LOF variation in 17 of our 27 LSL discovery genes (Fig. 2, Additional file 6: Table S5). Nearly half of these individuals (34/79 = 43%) also presented with CVM (Table 2). Conversely, among the 4778 unique case identifiers available for interrogation, 755 (15.8%) had phenotype accession data consistent with a cardiac phenotype; i.e., carriers of LOF variants in our discovery genes are four times more likely to have a cardiac phenotype among clinical cases (odds ratio (OR) = 4.03, 95% confidence interval (CI) 2.5–6.47). When we expanded this analysis to include algorithmically supported damaging variation in the same genes, however, there was no significant enrichment of these variants among cases with reported cardiac disease in their clinical requisition, which primarily reflects the referring diagnosis for clinical WES and not the full clinical phenotype.

In the clinical cohort, two candidate genes with observed de novo mutations from our primary analysis — *JARID2* and *SMURF1* — both had individual LOF carriers with CVM (Table 2), aortic and pulmonic stenosis with *JARID2* and dextrocardia with *SMURF1*. A missense mutation in *JARID2* was recently reported to segregate with BAV and a dilated aorta in a single family with LSLs [39]. In murine models, *Jarid2* is expressed throughout the embryonic heart and is necessary for normal cardiac development [40, 41] such that *Jarid2* null mice show cardiac defects including double outlet right ventricle and ventricular septal defects [42]. *SMURF1* (SMAD Specific E3 Ubiquitin Protein Ligase 1) encodes a downstream protein effector of transforming growth factor beta (TGFB) activity, which, in mouse models, is important to atrioventricular valve formation [43]. A de novo mutation in *SMURF1* was recently reported in a single individual with craniosynostosis [44]; no cardiac features were reported in the proband. Taken together, these results support a role for damaging variation in *JARID2* and *SMURF1* as causes of human CVM.

### Variant database annotation

Finally, within our samples we evaluated the prevalence of variants previously described as pathogenic in clinical databases. We first catalogued a priori candidate gene variants identified in LSL cases that were also represented in either the HGMD, 2015.1 or NCBI’s ClinVar. LSL cases presented 120 protein-altering variants in 55 distinct a priori candidate genes previously reported as pathogenic in these two databases (Additional file 7: Table S6). Evidence for pathogenicity was strongest at 29 sites that were extremely rare — 14 were case-exclusive and associated with human disorders related to cardiac development, and 15 were severely depleted across all comparison groups Minor Allele Count (MAC < 5). It is interesting to note that 14 of the latter variants were reported at a frequency higher than expected (MAF > 0.5%) in the ARIC comparison group, which was not ascertained for CVM; as a result, we do not consider these variants to be strong candidates for LSLs. However, we report our observed frequencies to inform future studies of pedigree segregation and functional models aimed at assessing the pathogenicity of the variants identified in our case cohort.

## Discussion

### Case-only cohort designs

The primary focus of our analysis was to identify likely pathogenic candidate variation in biologically plausible CVM genes occurring in individuals with a known CVM. We utilized a conservative, in silico criterion for definition for pathogenic variation alongside a broad bioinformatics-driven collation of genes with relevant biological and molecular function. We demonstrate that this approach can facilitate gene discovery for isolated, non-syndromic cardiac malformation. Novel candidate mutation in nine of our cases (2.6%) was confirmed to have arisen de novo*,* with inherited LOF mutations being observed in a further 15 patients (4.4%), inclusive of two patients with recessive or X-linked inheritance. In addition, 23 patients (6.7%) carried previously reported pathogenic variants for similar disease conditions involving CVM, which were also severely depleted in the comparison populations. In aggregate, WES of unrelated LSL cases identified candidate mutation in 49 (14.3%) of our LSL cases.

Our rare-case-only cohort design integrates well with current high-throughput sequencing pipelines since it utilizes bioinformatic support for prioritizing novel genes and filtering allele frequencies from large comparison cohorts and public databases. Improving integration with bioinformatic resources may allow for automated generation of a priori candidate genes for any rare condition. Moreover, such integration may be enabled further as a structured ontology, such as the Human Phenotype Ontology (HPO [45]) of clinical terms, is applied clinically and harmonization [11] of phenotype ontology between human, model organism, and biological pathway databases continues to improve. Clinical expertise, however, will remain crucial to further curating and refining candidate gene lists for patient care. Comparison of allele frequencies from large population-based resources remains a key aspect of assessing pathogenicity of known and novel candidate mutations, as low frequency variants can have distinct biological effects from higher frequency variants with similar functional annotation [46]. Combining this approach with disease segregation within pedigrees (particularly de novo variation), using Mendelian genomics and evidence for biological function relevant to the phenotype in question, brings our pipeline in line with recent clinical recommendations for inferring pathogenicity of genomic variants [47]. The rapid expansion of both clinical and bioinformatics resources thus bodes well for the future utility of case-only cohort screens for rare disease and suggests that this approach will continue to grow in power alongside population-based sequencing efforts.

### LSL gene discovery

By focusing on the intersection of case-exclusive LOF variation and an a priori candidate gene list, we identified novel candidate pathogenic variants in 7.9% (27/342) cases from our starting LSL cohort. Of the 27 resulting high-confidence genes implicated by intersection with our a priori gene list, interrogation of additional cases and clinical cohorts provided further evidence for LSL pathogenicity in nine genes; of these, *SMURF1*, *PLRG1*, and *ACVR1* have not been previously established in human LSLs or CHDs and emerged as the strongest novel potential disease gene candidates from our analyses. Integration of our candidate genes with individual disease cohorts and large collaborative projects that are ongoing in the USA and Europe holds the promise of confirming yet more of these candidates in the future. For example, our observation of *JARID2* variants in both our discovery and clinical cohorts adds to the previous single-family report in the literature [39] and strengthens arguments for *JARID2* as a *bona fide* LSL gene. Similarly, large-scale model organism knockout projects that include efforts to characterize developmental and cardiac phenotypes, such as the Knockout Mouse Phenotyping Program (KOMP2), have great potential to further facilitate the confirmation of putative human disease genes like those offered here. Lastly, expansion of annotated variation beyond LOF to include DNS variation will also aid disease gene discovery in CHDs.

### Complex mechanisms underlying non-syndromic LSLs

Our results also offer insights into the complex etiologies of abnormal cardiac development. First, virtually all our high-confidence pathogenic changes were in different genes and spanned different developmental cardiac gene pathways. This is reflective of the broader LSL literature, which has implicated several molecular pathways in the development of CHDs more generally and LSLs in particular [12, 39]. Further underscoring the complexity of LSLs, we also found evidence of additional modes of inheritance beyond the anticipated dominant inheritance, including both X-linked and recessive models in known syndromic genes. De novo dominant mutations have been reported to account for as many as 10% of CVM cases [13]. A general role for this genetic mechanism in CHDs is supported by our observation of de novo mutation in nine of the 342 (2.6%) of cases reported here. Our neonatal ascertainment of cases meant that we were limited in our ability to definitively exclude neurodevelopmental syndromes from our cohort; however, our results are broadly consistent with previous reports [48], intimating that de novo dominant mutation is less common among non-syndromic CHD cases and that the majority of rare LOF variants are inherited. We, and others, have previously established that up to 20% of parents and siblings of LSL probands will show subtle left-sided cardiac abnormalities including BAV and mitral valve leaflet redundancy, in which lesions can go undetected without specific cardiac imaging [49–51]. Despite this, cardiac imaging is not consistently undertaken in parents of children with LSLs, and we did not undertake systematic cardiovascular evaluation on parents recruited to our study; we are therefore unable to distinguish between incomplete penetrance and variable expressivity among the parent LOF carriers in our study. Nonetheless, our results confirm the notion that transmitted LOF (and DNS) variants observed in putative CHD candidates may yet contribute to cardiac developmental phenotypes across a phenotypic spectrum that includes clinically evident LSLs on the severe end of a spectrum of variably manifest CVMs. This adds to the emerging literature of complex interactions in cardiac and other congenital defects in which both *cis-*acting and *trans-*acting pathways can modify the expression of the disease [39, 44, 48, 52, 53]. Future studies in larger cohorts that are powered to systematically explore the complex potential mechanisms underlying this observation, including modifier genes and epistatic interactions, will be key to unraveling the complex genetic architecture of CHDs.

## Conclusions

Through a rigorous interrogation of known and suspected human CHD genes using available bioinformatic data resources, we have provided important insights into the genetic landscape of an important class of CHD. We find that the genetics underlying the development of LSLs, though complex and heterogeneous, is tractable in the context of large-scale databases, modern-day sequencing technologies, and carefully phenotyped clinical cohorts. This suggests that the expansion of international consortia and like collaboration could pay significant dividends for future studies addressing the most common class of birth defects.

## Additional files

Additional file 1: Table S1. Known LSL genes. Excel worksheet. (XLSX 11 kb)

Additional file 2: Table S2. Quantitative comparison of whole exome sequencing data from LSL discovery cohort and ARIC cohort. Excel worksheet. (XLSX 8 kb)

Additional file 3: Table S3. A priori gene list. Excel worksheet. (XLSX 110 kb)

Additional file 4: Figure S1. Distribution of rare sites within LSL cases. Adobe PDF. (PDF 208 kb)

Additional file 5: Table S4. Details of candidate variants identified in LSL cohort. Excel worksheet. (XLSX 12 kb)

Additional file 6: Table S5. Variants in candidate genes identified in clinical look-up cohort (Baylor Genetics). Excel worksheet. (XLSX 79 kb)

Additional file 7: Table S6. LSL cohort variants observed in ClinVar and/or HGMD. Excel worksheet. (XLSX 28 kb)

## Abbreviations

AS: Aortic valve stenosis
CoA: Coarctation of the aorta
CVM: Cardiovascular malformation
DNS: Damaging non-synonymous
HLHS: Hypoplastic left heart syndrome
IAAA: Interrupted aortic arch type A
LOF: Loss of function
LSL: Left-sided lesion
MA: MS, Mitral valve atresia and stenosis
SC: Shone’s complex
WES: Whole exome sequencing
