# Supplementary material for: Whole exome sequencing in 342 congenital cardiac left sided lesion cases reveals extensive genetic heterogeneity and complex inheritance patterns
Source: Genome Med. 2017 Oct 31;9:95. doi: 10.1186/s13073-017-0482-5 (PMC5664429; doi:10.1186/s13073-017-0482-5)
Supplement: Supplementary file 4 — Distribution of rare sites within LSL cases. Adobe PDF. (PDF 208 kb) [file 13073_2017_482_MOESM4_ESM.pdf]

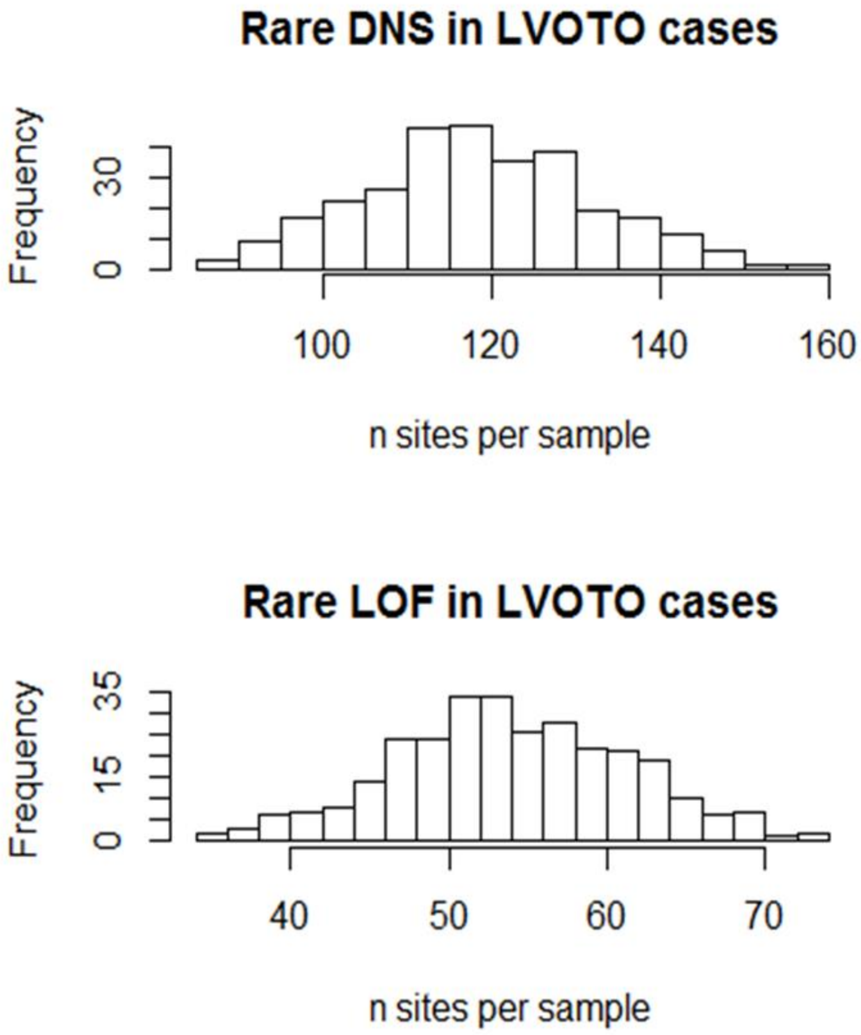

Figure S1 - Distribution of rare sites within LSL cases. These histograms depict the number of rare (a) DNS and (b) LOF sites per sample.
